# Wellen's Syndrome: Is One Electrocardiogram Good and Plenty?

**DOI:** 10.7759/cureus.4394

**Published:** 2019-04-05

**Authors:** Markayle R Schears, Bryan C Sleigh, Latha Ganti

**Affiliations:** 1 Emergency Medicine, University of Central Florida College of Medicine, Orlando, USA; 2 Emergency Medicine, Mercer University College of Medicine, Macon, USA; 3 Emergency Medicine, University of Central Florida College of Medicine / Hospital Corporation of America Graduate Medical Education (HCA GME) Consortium, Kissimmee, USA

**Keywords:** wellen's syndrome

## Abstract

The authors present a case of Wellen's syndrome, which has a characteristic T-wave on an electrocardiogram during a pain-free period in a patient with intermittent chest pain. The clinical presentation, pathophysiology, and management is discussed, and the importance of obtaining more than one electrocardiogram (ECG) is explained.

What this case adds to the literature is the fact that Wellen’s syndrome patients may present atypically with active chest pain and, as such, should be managed similarly to acute myocardial infarction patients. However, because the diagnosis of Wellen’s syndrome depends on an ECG obtained during the ensuing pain-free period, serial ECGs are usually required to reveal T-wave abnormalities in this context and have been shown to be disposition-altering in the Emergency Department (ED). Support for the death-denying outcome preferred in Wellen’s syndrome by patients and providers alike depends on recognizing the diagnosis and consulting cardiology expediently.

## Introduction

Wellen's syndrome (WS) captures the specific electrocardiogrphic (ECG) changes which indicate critical stenosis of the proximal left anterior descending (LAD) coronary artery. These ECG findings presage catastrophic myocardial infarction in patients presenting with unstable angina. The incidence of WS is estimated to be 10-15% of cases of acute coronary syndrome (ACS) [1] and 75% of these patients progress to myocardial infarction with medical management alone [2]. Timely identification of WS features correlates with a pre-infarction window that gives prepared clinicians the opportunity to intervene and prevent an impending infarction. This case highlights the importance of considering WS in one's differential, and to underscore that at least two ECGs are needed, as the ECG is paradoxically normal during the chest pain episode, but given standard treatment for ischemia, once the chest pain has resolved, ECG abnormalities characteristically appear during the pain-free period. Serial ECGs have a higher likelihood of capturing both this clinical paradox and uncovering what is most likely a relapsing and remitting phenomena reflective of unstable obstruction manifesting as T-wave abnormalities.

## Case presentation

A 51-year-old man with no history of coronary artery disease presented to the ED after he was awoken by sudden onset of left-sided chest pain. The pain escalated over 15 minutes. He described it as severe, radiating from the left anterior chest to his left scapula as well as down his left arm. The patient reported that he got out of bed and tried to "walk it off." At this time, he developed diaphoresis, nausea, and some light-headedness. He described his pain as 10/10 at its worst. His initial ECG was unremarkable. The patient was treated with aspirin, nitrates, and oxygen. His pain resolved within 15 minutes of arrival, or soon after vasodilators and anti-platelet therapy had been initiated. 

**Figure 1:**
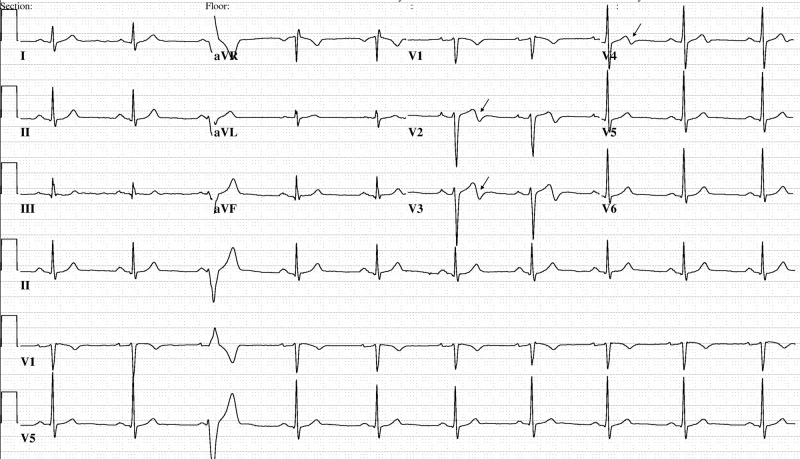
Electrocardiogram demonstrating biphasic T waves in leads V2, V3, and V4

**Figure 2:**
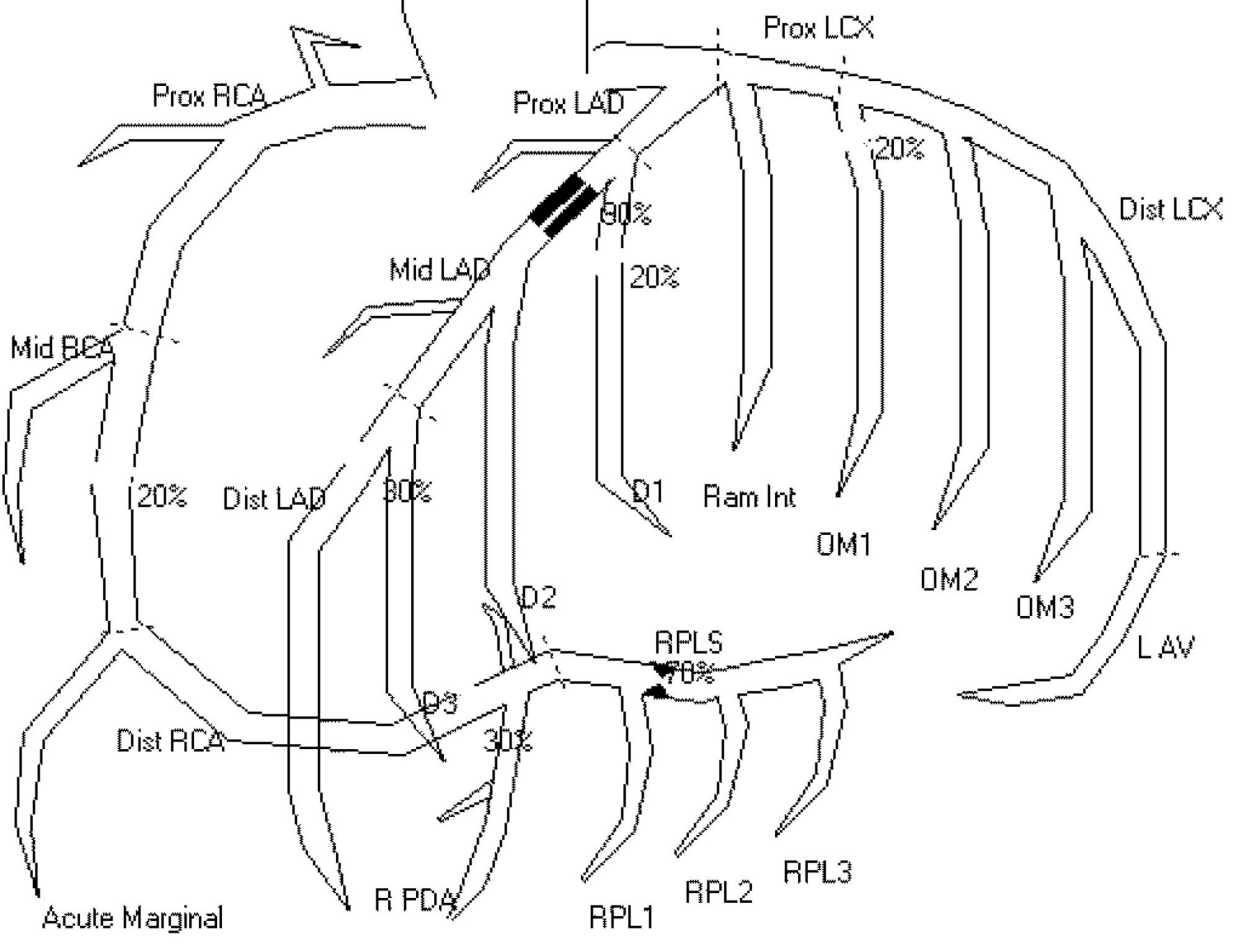
Location of lesion on cardiac catheterization. Prox-, Mid-, Distal LAD = proximal, middle and distal left anterior descending artery, Prox- and Distal LCX = proximal and distal left circumflex artery, LAV = left atrioventricular branch, Prox-, Mid-, Distal RCA = proximal, middle and distal right coronary artery, Ram Int = ramus interventricularis anterior (anterior interventricular branch of the left coronary artery), OM1,2,3,4 = obtuse marginal arteries (first through fourth), D1,2,3 = diagonal branches (first through third), RPDA = right posterior descending artery (runs in the posterior interventricular groove), RPLS = right posteriolateral segment artery; RPL1,2,3 (first through third) = right posterior lateral branches.

Repeat ECG (Figure 1) upon resolution of pain revealed biphasic T-waves in leads V2, V3, and V4, consistent with WS. The patient underwent urgent cardiac catheterization which did demonstrate a 90% proximal left anterior descending artery (LAD) lesion (Figure 2). The patient was stented and discharged the next day.

## Discussion

Wellen's syndrome was first identified in 1982 by de Zwaan et al. when they found a subgroup of admitted patients (14-18%) that shared a pattern of ECG changes that distinguished them from the sample population presenting with unstable angina [1-2]. Diagnostic criteria for WS comprise biphasic T-waves (24%) in anterior precordial leads V2-V3 or deeply inverted T-waves (76%), and although inverted T-waves can also be present in other precordial leads, the absence of ST-segment elevation and Q-waves across the precordium combined with normal R-wave progression are typical ECG manifestations [3-4]. Evolution of the pattern in the authors' experience invariably occurs: biphasic T-waves (Pattern A) in the distribution above become deeply and symmetrically inverted (Pattern B) which then extends to V4 then V5 then V6. This evolution was not described by de Zwaan and Wellens [2] probably because they did not do serial ECGs on admit patients nearly as often as we do today in the emergency department setting. Serial ECGs also demonstrate that T-wave abnormalities are persistent, remaining in place for hours to weeks, despite patients' reporting they are pain-free during this interval. Thus, these abnormalities do not resolve in the ED time frame, leading clinicians to suspect that WS may be an incidental reperfusion finding associated with occlusion of the LAD which itself is intermittent or reversible for a time. While the chances of capturing the transition from Pattern A -> Pattern B may increase with serial ECGs, there have been no good studies aimed at documenting this progression, and most likely because catheterization laboratory interventions with continuous ECG monitoring capabilities did away with the 'wait and see' approach of medical management for WS patients. History also reveals that anterior wall infarction is rapid, with a mean time of 6 to 8.5 days from the onset of WS [2]. A normal ECG with pain and an abnormal ECG without pain is a tip-off that may be missed without obtaining serial ECGs, especially in patients presenting with chest pain concurrent to their ED presentation. Although the WS diagnosis can be made from a single ECG to identify the T-wave abnormalities, most people that present to the ED with chest pain reflexively get an initial ECG to rule out ST segment elevation myocardial infarction; once the chest pain is resolved, another ECG may or may not be obtained to evaluate the patient's return to baseline status, to assess for silent ischemia, or to entertain a broader differential diagnosis. 

Further cardiac enzymes are normal to minimally elevated in WS, and as was true in this case; there were no ECG changes noted on ED arrival with chest pain, but this developed during the pain-free interval and after acute coronary syndrome (ACS) management was instituted. While two ECGs usually are good and plenty, one is not enough in patients presenting with ongoing chest pain and treated for unstable angina. Here, the first ECG with pain was normal and biphasic T-waves in V2, V3, and V4 rapidly emerged with repeat ECG obtained once the patient was pain-free, consistent with WS. Also note biphasic T-waves in V2, V3 distribution may persist or gradually progress to an inverted pattern with time, but most importantly, these abnormalities don't disappear during the ED stay. Because biphasic T-waves and inverted T-waves can be a normal variant, they may give providers a false sense of security and lead to premature closure in the case of a patient with recent but not concurrent chest pain, normal physical exam, normal cardiac enzymes, and potentially only one ECG. The literature reports on a Wellen’s patient discharged home from the ED after an initial ECG and cardiac enzymes were considered normal and a determination of low-risk for an adverse cardiac event was made, but two days later, the patient returned to the ED arriving in cardiac arrest from a massive myocardial infarction [5]. Reexamination of the only ECG from the prior ED visit showed biphasic T-waves in V2 and V3 that in hindsight were not normal. One wonders if serial ECGs in the ED might have captured the diagnosis of anterior descending T-wave syndrome or the progression in the changes. It also unknown whether activities of daily life (ADLs) with home disposition precipitated the acute event, which comparatively would have been avoided with hospitalization, given stress testing is contraindicated in Wellen’s syndrome patients. Moreover, cardiology consultation would have obtained timely coronary angiography and provided definitive intervention for a significant lesion.

The pathophysiology of ECG changes associated with WS is unclear. LAD obstruction causing intermittency or destabilized blood flow has also been hypothesized to cause local edema or stunning that may lead to the development of ECG changes in WS [6]. The differential for inverted T-waves includes cerebral hemorrhage, left ventricular hypertrophy, Takotsubo cardiomyopathy, pulmonary embolism, or edema; thus, proper clinical screening may be indicated for diagnostic accuracy.

Since WS is very specific for LAD stenosis [7] (sensitivity 69%, specificity 89%, positive predictive value 86%), stress testing is contraindicated, leaving urgent cardiac catheterization the only way to exclude critical LAD occlusion (pseudo-WS state differential listed above remains a diagnosis of exclusion) and keeps the door open for intervention: stents save lives when deployed for critical stenosis. A delay in diagnosis risks anterior wall myocardial infarction and for which non-invasive medical management is linked to death [2]. 

This case report adds to the known literature that Wellen's syndrome patients may present atypically with active chest pain and, as such, should be managed similarly to acute myocardial infarction patients. However, because the diagnosis of Wellen’s syndrome depends on an ECG obtained during the ensuing pain-free period, serial ECGs are usually required to reveal the T-wave abnormalities in this context and have been shown to be disposition-altering in the ED. Support for the death-denying outcome preferred in Wellen’s syndrome by patients and providers alike depends on recognizing the diagnosis and consulting cardiology expediently.

## Conclusions

Wellen's syndrome (also referred to as LAD coronary T-wave syndrome) consists of characteristic ECG findings in patients with unstable angina that are highly specific for critical LAD stenosis. The typical pattern includes biphasic T-waves in leads V2, V3, and V4. These T-wave abnormalities usually evolve into deeply inverted T-waves, and the pattern moves "down and out" extending across the precordium in an orderly fashion. Capturing T-wave changes in acutely asymptomatic patients is like finding trace evidence. It connotes a near miss with reperfusion artifact potentially the only detectable sign of a very precarious state: LAD preinfarction. Taking two ECGs to better inform the situation may save more lives than managing the case on the basis of just one ECG even when it is well-timed, because T-wave abnormalities can be subtle initially, incorrectly interpreted, or even missed. Maintaining a high level of suspicion and obtaining ECGs with and without chest pain in this case gave the emergency physician the additional insight needed to confidently proceed directly to cardiac catheterization as the lifesaving solution, best practice, and preferred clinical outcome. Thus, two ECGs are good and plenty, and not just one any more. 
